# Prognostic significance of long noncoding RNA TTN‐AS1 in various malignancies

**DOI:** 10.1002/cnr2.1876

**Published:** 2023-08-02

**Authors:** Guangyao Lin, Xiyu Liu, Chao Cong, Lianwei Xu

**Affiliations:** ^1^ Department of Gynecology, Longhua Hospital Shanghai University of Traditional Chinese Medicine Shanghai China

**Keywords:** biomarker, cancer, meta‐analysis, prognosis, TTN‐AS1

## Abstract

**Background:**

Increasing evidence has demonstrated that high TTN‐AS1 expression is highly related to poor prognosis in diverse human cancers. However, the findings concerning the prognostic value of TTN‐AS1 were inconsistent, as these conclusions were usually drawn with relatively small sample sizes. Hence, this meta‐analysis proposes to investigate the prognostic significance of TTN‐AS1 in multiple malignancies systematically.

**Methods:**

Web of Science, Springer, Embase, PubMed, Cochrane Library, and Scopus databases were comprehensively searched to retrieve studies related to the TTN‐AS1 expression with the prognosis of malignancies. The significance of the TTN‐AS1 in cancers was estimated by hazard ratios (HRs) or odds ratios (ORs). Additionally, the Gene Expression Profiling Interactive Analysis (GEPIA) analysis tool was used to strengthen our results further.

**Results:**

Twenty studies involving 17 different cancers and 1330 patients were recruited into this meta‐analysis. The research revealed that high TTN‐AS1 expression was remarkably associated with unfavorable overall survival (OS) (HR = 2.07, 95%CI [1.78, 2.41], *p* < .00001) when compared with low TTN‐AS1 expression in malignancies. Additionally, elevated TTN‐AS1 expression significantly contributed to lymph node metastasis (OR = 4.09, 95%CI [3.08, 5.44], *p* < .0001), larger tumor size (OR = 2.42, 95%CI [1.56, 3.77], *p* < .0001), worse tumor differentiation (OR = 0.36, 95%CI [0.22, 0.59], *p* < .0001) and more advanced tumor stage (OR = 0.29, 95%CI [0.22, 0.38], *p* < .0001) with low or no heterogeneity existing. Moreover, high TTN‐AS1 expression  was connected with worse disease‐free survival in five different cancers based on the GEPIA online database.

**Conclusions:**

The results of this meta‐analysis support that high TTN‐AS1 expression significantly correlates with worse prognosis in various cancers. Therefore, TTN‐AS1 may be considered as a novel biomarker for malignancies.

## 1 INTRODUCTION

Cancer is a critical cause of high mortality in every region globally, and there were nearly 10 million deaths due to cancer in 2020.^1^ Despite immense progress in the surveillance of cancers, such as serum markers circulating tumor DNA, circulating tumor cells, carcinoembryonic antigen, non‐invasive examination ultrasound, computed tomography (CT), magnetic resonance imaging, positron emission tomography (PET) and PET/CT, and so forth, the number of cancer cases still has not decreased over the years. According to a recent cancer statistic, there will be 28.4 million cancer cases in 2040.^2^ Therefore, the identification of  early and accurate cancer diagnostic biomarkers is essential for clinical diagnosis, therapy, risk assessment, and prognosis of cancer.^3^ However, it remains a challenge to detect cancers with ideal specific and sensitive biomarkers.^4^

Long non‐coding RNAs (lncRNAs) are described as RNAs that exceed 200 nucleotides in length and do not encode proteins.^5^ Simultaneously, lncRNAs, as novel RNAs discovered recently, have been proven to perform diverse cellular functions, including oncogenic or tumor suppressor functions.^6^ Titin‐antisense RNA1 (TTN‐AS1) is a newly identified lncRNA that maps to chromosome 2q31.2.^7^ Clinically, there is evidence suggesting that overexpression of TTN‐AS1 strongly associates with poor prognosis in numerous malignancies, such as digestive system tumors, respiratory tumors, reproductive system cancers, breast cancer, and other malignancies.^8^ Meanwhile, increased TTN‐AS1 expression is closely related to advanced tumor differentiation and tumor malignancy as well.^7^ In glioblastomas, for example, decreasing the expression of TTN‐AS1 could remarkably prolong the survival of mice and reduce the volume of tumor through promoting apoptosis and inhibiting the migration, proliferation, and invasion of glioblastoma cells, which may be correlate with the reduction of binding between miR‐320b and transcriptional factor EGR3 mRNA.^9^ Additionally, TTN‐AS1 was also revealed to accelerate epithelial‐mesenchymal transition (EMT), tumor angiogenesis, and cell cycle progression by downregulating miR‐320a, thus promoting the migration and proliferation of cholangiocarcinoma.^10^ In hepatocellular carcinoma, TTN‐AS1 could interact with miR‐139‐5p to modulate SPOCK1 expression, which then contributed to hepatocellular carcinoma cell multiplication, invasion, and EMT.^11^ Collectively, TTN‐AS1 might be a potential novel biomarker for cancer prognosis and therapeutic targets.

However, several clinical studies exploring the association of TTN‐AS1 expression with variables in malignancy have generated controversial conclusions. For example, Xiao et al.^12^ demonstrated that overexpression of TTN‐AS1 was related to high tumor grade, advanced tumor stage, vascular invasion, and lymph node metastasis (*p* < .05) but not to overall survival (OS) in bladder cancer. Lin et al.^13^ confirmed that high expression of TTN‐AS1 in patients with esophageal squamous cell carcinoma was significantly relevant to poor OS (*p* < .05) but not to the tumor stage (*p* > .05). Owing to the limitation of sample sizes from a single study, the significance of these associations may be inaccurately and insufficiently estimated. Therefore, this meta‐analysis was performed to further assess the prognostic importance of TTN‐AS1 expression in various malignancies.

## 2 MATERIALS AND METHODS

### 2.1 Search strategy

This study was conducted following the preferred reporting program of the systematic review and meta‐analysis (PRISMA).^14^ Web of Science, Springer, Embase, PubMed, Cochrane Library, and Scopus databases were thoroughly searched for eligible studies from inception until January 22, 2023. The search terms were as follows: “long non‐coding RNA TTN‐AS1” OR “titin‐antisense RNA1” OR “TTN‐AS1” OR “lncRNA TTN‐AS1.” Besides, the references of retrieved studies were evaluated carefully to look for more potentially relevant articles.

### 2.2 Inclusion and exclusion criteria

A relevant study satisfying the following inclusion criteria would be included in the analysis: (1) Original research articles; (2) study focused on the associations between various human malignancies and TTN‐AS1 expression; (3) study reported OS, disease‐free survival (DFS), or clinic parameters at least; (4) study's sample was derived from tissue; (5) study involved sufficient data; (6) there were no ethnic and geographical restrictions.

The exclusion criteria were as listed: (1) Duplicate publications, cellular‐based experiments, reviews, letters, and animal experiments; (2) study data acquired from public databases; (3) articles with insufficient data.

### 2.3 Data extraction and quality assessment

The full text of the relevant studies that met the inclusion criteria were selected for the data collection process. The major characteristics of the included studies were summarized according to a predesigned standardized template: the author's last name, publication time, cancer types, sample capacity, the number of patients based on TTN‐AS1 expression level, detection methods, outcomes (e.g., OS and DFS), hazard ratio (HR) and corresponding 95% confidence interval (CI) of OS, OS data extraction methods and follow‐up time. If the study failed to offer the HR and 95% CI in the article, Engauge Digitizer 4.1 software was adopted to extrapolate the HR and 95% CI indirectly, based on the available Kaplan–Meier curves.^15^ Furthermore, the Newcastle–Ottawa scale (NOS) was adopted to estimate the quality of the included studies. The NOS scores ranged from 0 to 9. When an article scored greater than or equal to 6, it was considered to be of high‐quality.^16^

### 2.4 DFS in bioinformatics database

Gene Expression Profiling Interactive Analysis (GEPIA), a public database from TCGA and GTEx data (http://gepia.cancer-pku.cn/), was applied to evaluate the correlation of TTN‐AS1 expression level with DFS. We input the gene name “TTN‐AS1” to match 33 types of cancers in GEPIA. A median was adopted for the cut‐off value. *p* < .05 was considered significantly statistical.

### 2.5 Statistical analysis

EndNote 20.2 software was adopted for data management. Stata 15.1 and Review Manager 5.3 software were performed for statistical analysis. The prognosis of various malignancies was pooled as HR value with 95% CI. Additionally, clinicopathologic parameters were detected as odds ratio (OR) value with 95% CI. The interstudy heterogeneity was assessed with *I* ^2^ statistics. An *I* ^2^ ≤ 50% indicated no statistically significant heterogeneity. The fixed‐effect model ought to be applied. If *I* ^2^ > 50%, it represents a substantial heterogeneity among the included publications; as a result, the random‐effect model should be adopted. The forest plot was generated by taking advantage of Review Manager 5.3 software. The publication bias was evaluated by Begg's and Egger's tests, and the robustness of analysis outcomes was estimated by sensitivity analysis through omitting individual studies.

## 3 RESULTS

### 3.1 Included articles

**FIGURE 1:**
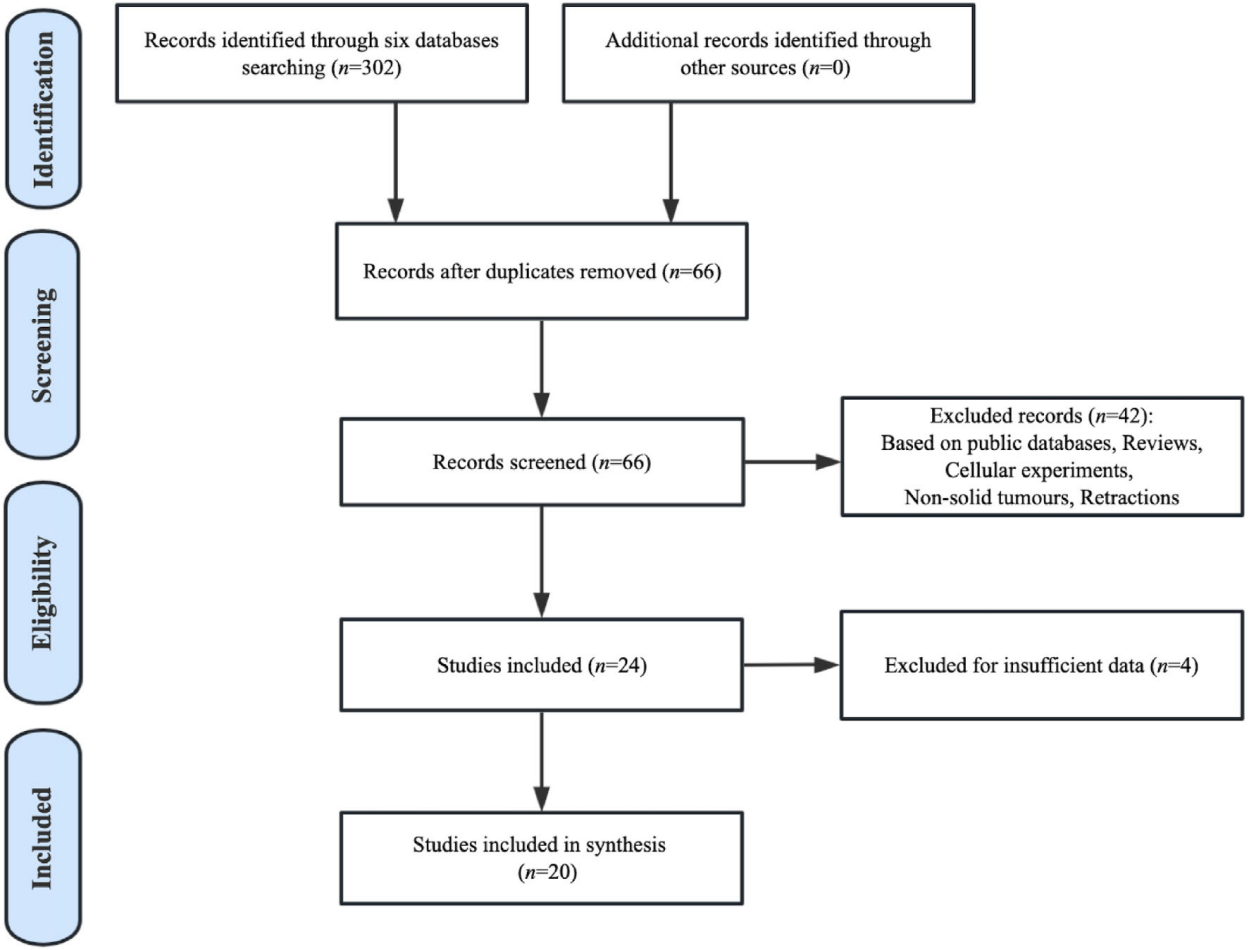
Paper selection flowchart.

A total of 302 articles were initially retrieved. After removing 236 studies owned to duplication, 66 studies remained for further evaluation. Subsequently, 42 studies were removed based on eligibility criteria through reading titles, abstracts, or full text. As for the remaining 24 studies, 4 with insufficient data were also excluded. Ultimately, 20 studies published between 2018 and 2021 were included in this analysis (Figure 1).

### 3.2 Study characteristics

A total of 20 studies containing 1330 cancer patients were included in this meta‐analysis. All included studies were from China. Quantitative real‐time polymerase chain reaction (qRT‐PCR) was performed to detect TTN‐AS1 expression in tissues. The sample sizes varied from 36 to 145. There were 718 cases in the high TTN‐AS1 expression group and 612 cases in the low TTN‐AS1 expression group. Moreover, all included studies provided clinicopathologic parameters, including lymph node metastasis, age, tumor size, gender, tumor differentiation, and tumor stage. Twelve studies reported OS, and one study reported DFS. There were 17 different types of malignant tumors in this meta‐analysis. Regarding the study quality, all included publications scored ≥6, attesting to the high quality of the 20 studies (Table 1).

**TABLE 1:** Main characteristics of included study.

Author	Year	Cancer type	Sample size (*n*)	High expression (*n*)	Low expression (*n*)	Detection method	Outcome	HR (95% CI) for OS	Data extraction method	Follow‐up (m)	NOS
Shen^17^	2020	EC	45	30	15	qRT‐PCR	CP, OS	1.93 (0.67–5.57)	Indirectly	40	7
Chen^18^	2018	CC	45	23	22	qRT‐PCR	CP, OS	2.82 (1.14–6.96)	Indirectly	60	6
Jin^19^	2021	OSCC	36	18	18	qRT‐PCR	CP	NA	Indirectly	NA	8
Xiao^12^	2021	BLC	130	95	35	qRT‐PCR	CP, OS, DFS	2.14 (0.03–142.21)	Indirectly	80	7
Fang^20^	2020	BRC	40	24	16	qRT‐PCR	CP, OS	2.22 (0.34–14.61)	Indirectly	120	7
Feng^21^	2020	BRC	56	28	28	qRT‐PCR	CP	NA	Indirectly	NA	7
Zhu^10^	2020	CCA	39	21	18	qRT‐PCR	CP	NA	Indirectly	NA	9
Cui^22^	2019	CLC	95	50	45	qRT‐PCR	CP, OS	1.54 (0.7–3.36)	Indirectly	60	7
Lin^13^	2018	ESCC	58	29	29	qRT‐PCR	CP, OS	2.73 (1.27–4.58)	Directly	94	9
Lin^23^	2020	ccRCC	145	65	80	qRT‐PCR	CP	NA	Indirectly	NA	6
Zhong^24^	2019	LA	74	42	32	qRT‐PCR	CP, OS	1.68 (0.35–8.06)	Indirectly	60	7
Qi^25^	2020	SCLC	50	25	25	qRT‐PCR	CP	NA	Indirectly	NA	7
Wang^26^	2019	CLC	40	13	27	qRT‐PCR	CP, OS	1.61 (0.57–4.52)	Indirectly	40	8
Li^27^	2019	OS	47	24	23	qRT‐PCR	CP, OS	1.74 (0.63–4.81)	Indirectly	60	8
Meng^28^	2021	OS	63	32	31	qRT‐PCR	CP	NA	Indirectly	NA	8
Zhao^29^	2021	PC	78	45	33	qRT‐PCR	CP	NA	Indirectly	NA	6
Cui^30^	2019	PTC	92	49	43	qRT‐PCR	CP, OS	1.98 (0.69–5.71)	Indirectly	60	6
Zhu^11^	2021	HCC	70	35	35	qRT‐PCR	CP	NA	Indirectly	NA	8
Chang^31^	2020	GL	45	30	15	qRT‐PCR	CP, OS	2.38 (0.83–6.81)	Indirectly	45	7
Dong^32^	2019	GC	82	40	42	qRT‐PCR	CP, OS	2.28 (0.74–7.07)	Indirectly	60	6

Abbreviations: BLC, bladder cancer; BRC, breast cancer; CC, cervical cancer; CCA, cholangiocarcinoma; ccRCC, clear cell renal cell carcinoma; CI, confidence interval; CLC, colorectal cancer; CP, clinicopathologic parameters; DFS, disease‐free survival; EC, endometrial cancer; ESCC, esophageal squamous cell carcinoma; GC, gastric cancer; GL, glioma; HCC, hepatocellular carcinoma; HR hazard ratio; LA, lung adenocarcinoma; NA, not available; NOS, Newcastle‐Ottawa Scale; OS, osteosarcoma; OS, overall survival; OSCC, oral squamous cell carcinoma; PC, pancreatic cancer; PTC, papillary thyroid cancer; qRT‐PCR, quantitative real‐time reverse transcription polymerase chain reaction; SCLC, non‐small cell lung cancer.

### 3.3 Association between TTN‐AS1 expression and clinical covariates

**FIGURE 2:**
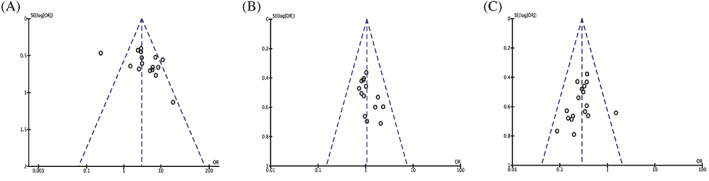
The funnel plots for lymph node metastasis (A), gender (B), and tumor stage (C).

The meta‐analysis explored the connection between clinicopathologic parameters and TTN‐AS1, as shows in Table 2. Four studies^17^, ^18^, ^20^, ^21^ in which cases were all female, were excluded when analyzing the covariate of gender. The results indicated that high TTN‐AS1 expression was noticeably associated with lymph node metastasis (*p* < .0001), larger tumor size (*p* < .0001), worse tumor differentiation (*p* < .0001), and more advanced tumor stage (*p* < .0001). Nevertheless, TTN‐AS1 expression was not evidently associated with age (*p* = .68) and gender (*p* = .69). As for the publication biases, we performed funnel plot analysis for these metrics containing more than 10 studies, and no apparent asymmetry was observed (Figure 2).

**TABLE 2:** The analysis of the relationship between TTN‐AS1 expression and clinical covariates.

Covariates	Studies (*n*)	Case (*n*)	OR 95% CI	*p*	*I* ^2^ (%)	Model
Lymph node metastasis (Yes vs. No)	15	924	4.09 [3.08, 5.44]	<.0001	8	Fixed
Age (≥60 vs. <60)	8	487	1.08 [0.75, 1.57]	.68	0	Fixed
Tumor size (≥5 cm vs. <5 cm)	6	346	2.42 [1.56, 3.77]	<.0001	42	Fixed
Gender (Male vs. Female)	14	1039	1.05 [0.82, 1.36]	.69	0	Fixed
Tumor differentiation (Well vs. Moderate/poor)	7	316	0.36 [0.22, 0.59]	<.0001	38	Fixed
Tumor stage (I–II vs. III–V)	17	1127	0.29 [0.22, 0.38]	<.0001	0	Fixed

### 3.4 Association between TTN‐AS1 expression and OS

**FIGURE 3:**
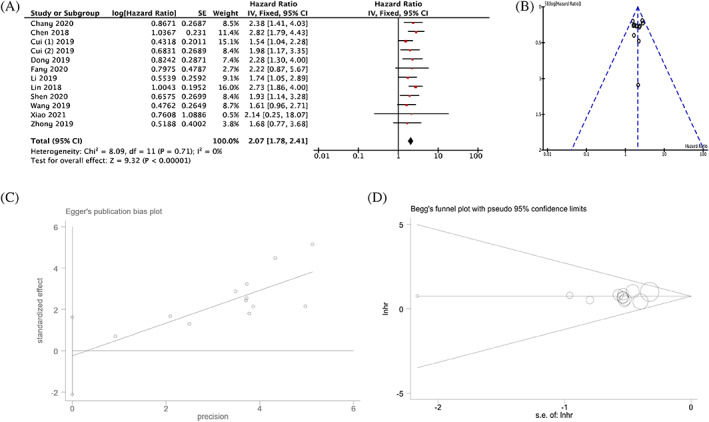
The forest plot (A); the funnel plot (B), the Egger's test (C) and Begg's test (D) for the association between TTN‐AS1 expression levels with OS.

Twelve studies involving 793 patients were extracted in this meta‐analysis for OS. As there was no heterogeneity existing among these studies (*I* ^2^ = 0), the fixed‐effect model was applied and the result revealed that high TTN‐AS1 expression was prominently correlated with shorter OS while compared with low TTN‐AS1 expression (HR = 2.07, 95%CI [1.78, 2.41], *p* < .00001, Figure 3A). As for the publication biases among the included studies, no apparent asymmetry was observed based on funnel plots (Figure 3B), and Begg's and Egger's tests also proved that there was no publication bias among studies (*p* > .05) (Figure 3C,D). The pooled results were robust after verifying by sensitivity analysis. In addition, subgroup analysis was carried out depending on the cancer types and sample capacity. As shown in Table 3, high TTN‐AS1 expression indicated worse OS than low TTN‐AS1 expression in the two subgroup analysis, regardless of cancer types and sample capacity (*p* < .0001).

**TABLE 3:** The subgroup analysis the relationship between TTN‐AS1 expression and OS.

Category	Studies (*n*)	Case (*n*)	HR 95% CI	*p*	*I* ^2^ (%)	Model
*Cancer types*						
Gynecologic cancer	3	130	2.38 [1.72, 3.29]	<.0001	0	Fixed
Others	9	663	1.99 [1.67, 2.37]	<.0001	0	Fixed
*Sample capacity*						
≥50	6	531	2.01 [1.60, 2.54]	<.0001	0	Fixed
<50	6	262	2.09 [1.68, 2.61]	<.0001	0	Fixed

### 3.5 DFS in the GEPIA database

**FIGURE 4:**
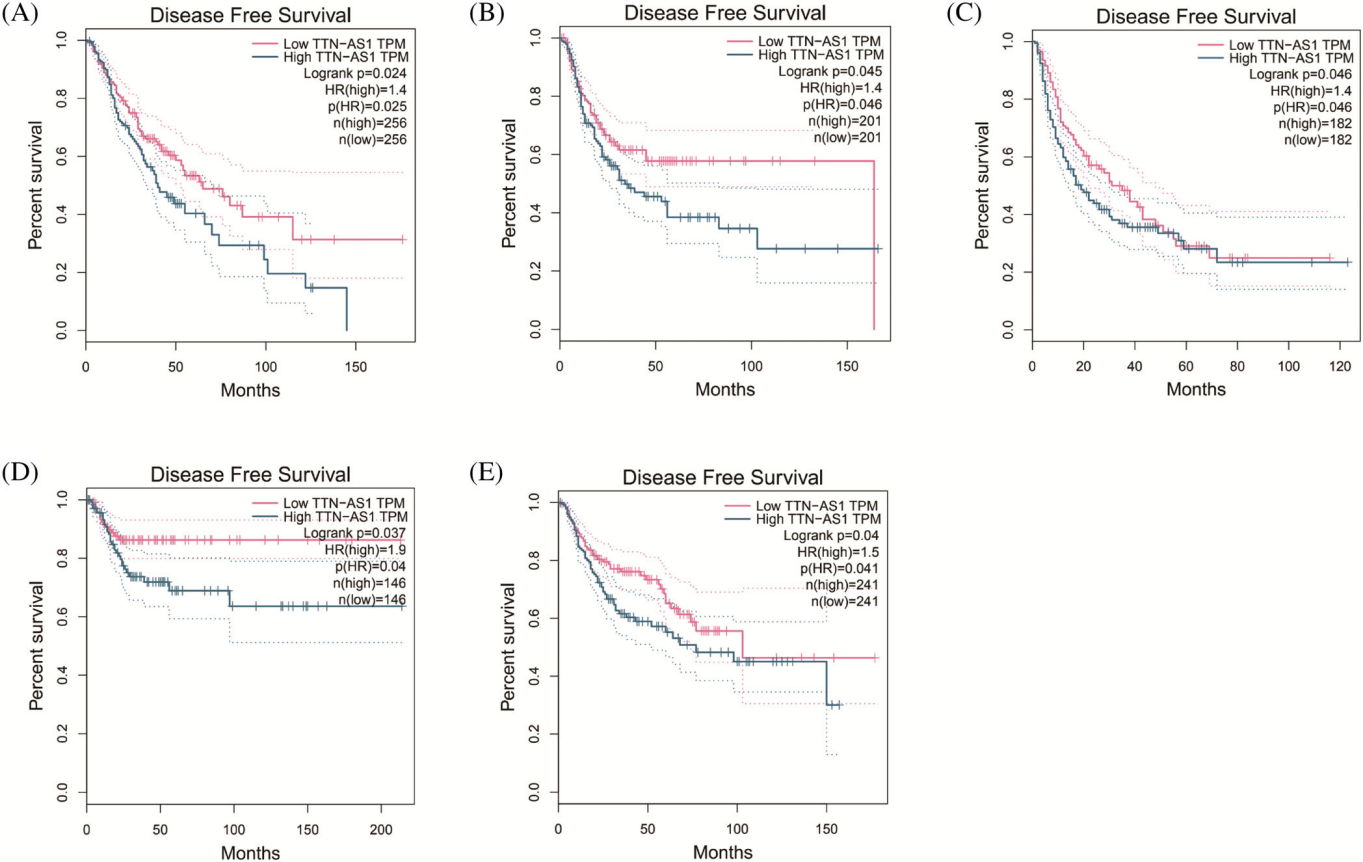
Supplement of the prognostic value of TTN‐AS1 in Gene Expression Profiling Interactive Analysis (GEPIA) database. (A) Disease‐free survival (DFS) plots of TTN‐AS1 in brain lower grade glioma (LGG); (B) DFS plots of TTN‐AS1 in bladder urothelial carcinoma (BLCA); (C) DFS plots of TTN‐AS1 in liver hepatocellular carcinoma (LIHC); (D) DFS plots of TTN‐AS1 in cervical squamous cell carcinoma and endocervical adenocarcinoma (CESC); (E) DFS plots of TTN‐AS1 in lung squamous cell carcinoma (LUSC).

Although DFS is an essential prognostic index for patients with malignancy, only one study reported DFS.^12^ Therefore, we used the GEPIA dataset to determine the expression of TTN‐AS1 in five kinds of related malignancies, including brain lower grade glioma, bladder urothelial carcinoma, liver hepatocellular carcinoma, cervical squamous cell carcinoma and endocervical adenocarcinoma, and lung squamous cell carcinoma. We found that high TTN‐AS1 expression showed a worse DFS than low TTN‐AS1 expression (*p* < .05), as listed in Figure 4. This result verified that the TTN‐AS1 expression is overexpressed in various malignancies and negatively correlated with the DFS of these patients with malignancy.

## 4 DISCUSSION

The aberrant expression of numerous lncRNAs, demonstrated recently, might interrupt various cancer progression and metastasis by affecting  the coding genes' expression and splicing.^33^ Meanwhile, lncRNAs are also highly involved in tumor initiation and invasiveness by regulating the process of EMT and mesenchymal‐epithelial transition.^34^ Increasing studies have suggested that TTN‐AS1 was dysregulated in various malignancies, and many have evaluated the prognostic significance of TTN‐AS1 expression in tumor tissues, but with conflictive conclusions. Therefore, this meta‐analysis was performed to investigate the clinical significance of TTN‐AS1 expression systematically.

This study demonstrated that increased TTN‐AS1 expression was explicitly associated with shorter OS and worse DFS. The close correlation between TTN‐AS1 expression and OS and DFS was validated by subgroup analysis and the GEPIA database, respectively. Furthermore, we also found that high TTN‐AS1 expression rendered more advanced clinical stage, lymph node metastases, larger tumor size, and worse tumor differentiation when compared with low TTN‐AS1 expression. Taken together, our findings suggested that increased TTN‐AS1 expression implied a worse prognosis, indicating that TTN‐AS1 expression might be served as a promising biomarker for prognosis in various cancers.

In addition, the mechanisms underlying the TTN‐AS1 expression in malignancies are rather complex. Chen et al. found that TTN‐AS1 could expedite the proliferative, migratory, and invasive capabilities of nasopharyngeal carcinoma by targeting miR‐876‐5p/UPF1/NETO2 signaling.^35^ Besides, TTN‐AS1 exerted its carcinogenic influence via facilitating TTN promoter activity in the nucleus and the accumulation of TTN in the cytoplasm, and animal experiments also found high TTN‐AS1 expression tended to damage the lung and kidney of skin cutaneous melanoma mice models.^36^ Similarly, Jia et al. observed that TTN‐AS1 contributed to the migration and invasion of lung adenocarcinoma by adjusting the TTN‐AS1/miR‐142‐5p/CDK5 axis.^37^ TTN‐AS1 also weakened the inhibition of apoptosis of hepatocellular carcinoma by sponging miR‐16‐5p expression, regulating the PTEN/Akt signaling pathway, and enhancing cyclin E1 expression.^38^ Another study indicated that inhibiting TTN‐AS1 expression could accelerate apoptosis of glioblastoma cells and suppress its malignant biological behaviors, like proliferation, migration, and invasion via the miR‐320b/EGR3/PKP2 axis.^9^ In prostatic cancer, TTN‐AS1 could negatively interact with miR‐193a‐5p, which then adjusted the protein levels of p21, Bax, CyclinD1, and Bcl‐2 to involve in cell apoptosis and proliferation.^39^ In gastric cancer, high TTN‐AS1 expression contributed to the poor OS and tumor progression by the TTN‐AS1‐miR‐376b‐3p‐KLF12 axis.^32^ Moreover, some investigations also found high expression of TTN‐AS1 could modulate a large number of proteins associated with tumor invasiveness, proliferation, and dissemination; nevertheless, they failed to elaborate further on how TTN‐AS1 impacts this results through tumor‐associated signaling pathways according to in vivo or in vitro experiments.^40^, ^41^ Therefore, further investigation is necessary to verify the underlying mechanisms.

To the best of our knowledge, this is the first meta‐analysis to evaluate the prognosis significance of TTN‐AS1 in diverse malignancies. Although the potential role of TTN‐AS1 in various cancers has been proved recently, firm conclusions need to be drawn due to the limited sample sizes. Hence, this meta‐analysis, which included 20 studies involving 17 types of cancer and 1330 patients, aimed to provide more convincing evidence. Interestingly, our findings indicated that high TTN‐AS1 expression was significantly associated with worse OS, and Begg's tests, Egger's tests, along with sensitivity analysis results demonstrated that our conclusion was reliable and robust. Second, two studies failed to provide the correlation of tumor differentiation with TTN‐AS1 expression (*p* > .05),^17^, ^32^ which was contrary to this meta‐analysis. Third, another difference was that our study indicated a significant association of TTN‐AS1 with tumor stage, which was not appreciated in one study.^10^ Fourth, another study did not support larger tumor size was related to high TTN‐AS1 expression,^27^ which had also been rebutted by the pooled results. Last, subgroup analysis and funnel plot of OS were performed, and the results, with no heterogeneity, firmly supported the prognostic  significance of measuring and interpreting TTN‐AS1 in various malignancies. Therefore, our meta‐analysis suggested that TTN‐AS1 expression could be a valuable predictive biomarker for prognostic value in multiple malignancies.

Several limitations should be emphasized for the results of our meta‐analysis. First, the HRs in most of the included studies were extracted indirectly by reconstructing survival curves using the available software introduced by Tierney et al.,^12^ which might inevitably lead to subjective errors. Second, all included studies were from China, which might make our conclusions not generalizable outside of China. Third, the postsurgical treatment modality might also be an essential factor in the prognosis of cancer patients. Nevertheless, these clinical data were inaccessible in the included studies, which might be an inherent deficiency of our meta‐analysis. Fourth, although only one study obplease served the expression of TTN‐AS1 in DFS, we used the GEPIA database to compensate for this limitation. Last, since most of the publication has different cut‐off value for tumor size and patient age, this had resulted in smaller pooled studies for these two covariates. However, we selected the cut‐off values most commonly used in the literature for this meta‐analysis. Therefore, more high‐quality studies conducted in diverse regions worldwide are necessary to address these limitations in the short future.

## 5 CONCLUSION

High TTN‐AS1 expression significantly correlates with shorter OS, adverse DFS, lymph node metastases, larger tumor size, worse differentiation, and more advanced tumor stage when compared with low TTN‐AS1 expression. TTN‐AS1 may be used as a novel biomarker for malignancies.

## AUTHOR CONTRIBUTIONS

**Guangyao Lin:** Conceptualization (equal); formal analysis (equal); methodology (equal); validation (equal); writing – original draft (equal); writing – review and editing (equal). **Xiyu Liu:** Methodology (equal); resources (equal); software (equal). **Chao Cong:** Data curation (equal); investigation (equal); validation (equal); visualization (equal). **Lianwei Xu:** Conceptualization (equal); supervision (equal); writing – review and editing (equal).

## CONFLICT OF INTEREST STATEMENT

The authors have stated explicitly that there are no conflicts of interest in connection with this article.

## ETHICS STATEMENT

This article does not contain any studies with human participants or animals performed by any of the authors.

## Data Availability

The data sets that were used and/or analyzed during the present study are available from the first or corresponding author.
